# *LARGE* expression in different types of muscular dystrophies other than dystroglycanopathy

**DOI:** 10.1186/s12883-018-1207-0

**Published:** 2018-12-15

**Authors:** Burcu Balci-Hayta, Beril Talim, Gulsev Kale, Pervin Dincer

**Affiliations:** 1 Department of Medical Biology, Hacettepe University Faculty of Medicine, 06100 Sihhiye, Ankara, Turkey; 2 Department of Pediatrics, Pathology Unit, Hacettepe University Faculty of Medicine, 06100 Sihhiye, Ankara, Turkey

**Keywords:** *LARGE*, Alpha-dystroglycan, Hypoglycosylation, Muscular dystrophy, Skeletal muscle

## Abstract

**Background:**

Alpha-dystroglycan (αDG) is an extracellular peripheral glycoprotein that acts as a receptor for both extracellular matrix proteins containing laminin globular domains and certain arenaviruses. An important enzyme, known as Like-acetylglucosaminyltransferase (LARGE), has been shown to transfer repeating units of -glucuronic acid-β1,3-xylose-α1,3- (matriglycan) to αDG that is required for functional receptor as an extracellular matrix protein scaffold. The reduction in the amount of LARGE-dependent matriglycan result in heterogeneous forms of dystroglycanopathy that is associated with hypoglycosylation of αDG and a consequent lack of ligand-binding activity. Our aim was to investigate whether LARGE expression showed correlation with glycosylation of αDG and histopathological parameters in different types of muscular dystrophies, except for dystroglycanopathies.

**Methods:**

The expression level of *LARGE* and glycosylation status of αDG were examined in skeletal muscle biopsies from 26 patients with various forms of muscular dystrophy [Duchenne muscular dystrophy (DMD), Becker muscular dystrophy (BMD), sarcoglycanopathy, dysferlinopathy, calpainopathy, and merosin and collagen VI deficient congenital muscular dystrophies (CMDs)] and correlation of results with different histopathological features was investigated.

**Results:**

Despite the fact that these diseases are not caused by defects of glycosyltransferases, decreased expression of *LARGE* was detected in many patient samples, partly correlating with the type of muscular dystrophy. Although immunolabelling of fully glycosylated αDG with VIA4–1 was reduced in dystrophinopathy patients, no significant relationship between reduction of *LARGE* expression and αDG hypoglycosylation was detected. Also, Merosin deficient CMD patients showed normal immunostaining with αDG despite severe reduction of *LARGE* expression.

**Conclusions:**

Our data shows that it is not always possible to correlate *LARGE* expression and αDG glycosylation in different types of muscular dystrophies and suggests that there might be differences in αDG processing by LARGE which could be regulated under different pathological conditions.

## Background

Dystroglycan (DG), a heterodimeric transmembrane glycoprotein, is a central component of the dystrophin-glycoprotein complex (DGC) and responsible for a variety of physiological and developmental processes, including muscle stabilization, basement membrane assembly, cell migration and signalling [1]. The protein is synthesized as a precursor molecule that is post-translationally cleaved into a cell surface α- and a transmembrane β- subunits [2]. The extracellular subunit, alpha-dystroglycan (αDG), is a highly glycosylated basement membrane protein that acts as a receptor for non-collagenous proteins in the extracellular matrix (ECM) containing laminin globular domains, and for Old World arenaviruses [1, 3–5]. Mutations in the DG-encoding gene *(DAG1)* [6, 7] and several other genes whose products are involved, directly or indirectly, in glycosylation pathway of αDG [8] have been identified in various forms of congenital and limb-girdle muscular dystrophies (CMD/ LGMDs). These disorders are all associated with hypoglycosylation of αDG and a consequent lack of ligand-binding activity and thus are collectively termed as dystroglycanopathies [9].

Although the precise glycosylation pathway of αDG is not fully understood, the function of Like-acetylglucosaminyltransferase (LARGE) enzyme has been of particular interest. It is encoded by one of the largest genes (*LARGE)* in the human genome [10–12], which is mutated in the myodystrophy mouse (LARGE-myd) [10, 13] and in patients affected by MDC1D, a subclass of CMD associated with skeletal muscle and structural brain involvement [11]. The mentioned enzyme is a bifunctional glycosyltransferase, with both xylosyltransferase and glucuronyltransferase activities and plays an important role in the glycosylation pathway of αDG. It transfers a novel heteropolysaccharide structure, repeating units of -glucuronic acid-β1,3-xylose-α1,3- to the basement membrane receptor DG [14]. This structure has been recently termed as matriglycan, which is bound to the αDG through a phosphorylated *O*-mannosyl glycan anchored at Thr317 and Thr319 in the mucin-like domain [8, 15, 16]. It is required for the αDG to bind laminin-G domain-containing ECM ligands including laminin, agrin, perlecan and neurexin [4, 17–19]. It has been reported that LARGE-dependent matriglycan structure plays an important role for normal skeletal muscle function and the consequent reduction in the amount of glycans on αDG causes structural alterations of the basement membrane, immature neuromuscular junctions and dysfunctional muscle predisposed to dystrophy [20]. Uniquely, transient overexpression of *LARGE* has been shown to increase glycosylation of αDG as evaluated by increased immunoreactivity to antibodies IIH6 and VIA4–1 both of which are known to recognize carbohydrate moieties and leads to a recovery of receptor function in cells derived from patients diagnosed as Fukuyama CMD (FCMD), muscle-eye-brain disease (MEB) and Walker–Warburg syndrome (WWS) [17]. Also, the identical results has been reported in vivo, after adenovirus or adeno-associated virus mediated gene transfer of *LARGE* in *Fukutin (FKTN), Fukutin-related protein (FKRP)* and *Protein-O-mannose ß2-N-acetylglucosaminyltransferase 1 (POMGNT1)* deficient mice models [21–23]. Although a recent study demonstrates that fukutin is required for the ability of LARGE to hyperglycosylate αDG [24], this strategy is already regarded as a promising therapeutic approach for preventing/slowing progression of a broad range of dystroglycanopathies regardless of the causative gene defects. However, it remains crucial to analyze such a strategy in different types of muscular dystrophies other than dystroglycanopathy.

In this study, we analyzed the influence of *LARGE* gene expression profile on VIA4–1 immunoreactivity in different types of muscular dystrophies, except for dystroglycanopathies [i.e. Duchenne muscular dystrophy (DMD), Becker muscular dystrophy (BMD), sarcoglycanopathy, dysferlinopathy, calpainopathy, and merosin and collagen VI deficient CMDs]. Correlation between different histopathological features, *LARGE* expression profile and glycosylation status of αDG was investigated. We detected reduced expression level of *LARGE* in different types of muscular dystrophies, partly correlating with the severity of dystrophic changes, but we did not find any significant relationship between reduction of *LARGE* expression and VIA4–1 immunoreactivity.

## Methods

### Patients and muscle biopsies

The study protocol was approved by the Hacettepe University Faculty of Medicine Ethical Review Board (FON 10/35–11). As the legal age of consent is 18 years of age in Turkey, written informed consent was obtained from patients’ parents or legal guardians at the time of diagnostic muscle biopsy. Skeletal muscle biopsies from 3 controls and 26 patients with clinicopathological and/or molecular diagnosis of various forms of muscular dystrophy other than dystroglycanopathy were included in this study. The number of patients in each disease type is indicated in parentheses; DMD (3), BMD (4), sarcoglycanopathy (5), dysferlinopathy (1), calpainopathy (1), Merosin deficient CMD (4), Collagen VI deficient CMD (3), unclassified CMD (2), and unclassified MD (3). All patients have been investigated in the Department of Pediatrics at Hacettepe University Faculty of Medicine and open biopsies were taken from vastus lateralis muscle at the time of diagnosis. The muscle biopsy specimens were rapidly frozen in isopentane cooled in liquid nitrogen and kept at − 80 °C until use. Serial transverse muscle sections were cut by cryostat for histochemical staining. Standard histological and histochemical techniques were applied to cryostat sections, including Haematoxylin and eosin (H&E), modified Gomori trichrome, periodic acid Schiff, oil-red-O, nicotinamide adenine dinucleotide dehydrogenase-tetrazolium reductase, succinic dehydrogenase, cytochrome-c oxidase and ATPase. Immunohistochemical and/or immunofluorescent studies were done at the time of diagnosis, using antibodies against dystrophins, sarcoglycans, dysferlin, laminin α2 (merosin), collagen VI, and αDG, as appropriate.

### Histopathological evaluation

H&E stained archive sections were examined to identify the severity of various dystrophic changes, including necrosis, regeneration, adiposis, endomysial and perimysial fibrosis and inflammation. These pathological changes were scored from 0 (absent) to 4 (severe).

### Immunofluorescent staining

For immunofluorescent staining, skeletal muscle cryosections of 7 μm were immunostained with primary antibodies [1/10 for mouse anti-α-Dystroglycan antibody (clone VIA4–1 against fully glycosylated form of αDG, Millipore), 1/50 for sheep anti-α-Dystroglycan core antibody (R&D Systems: AF6868), 1/100 for mouse anti-Spectrin (NCL-SPEC1 Clone: RBC2/3D5, Novocastra), 1:20 for anti-Beta-Dystroglycan (βDG) (NCL-b-DG Clone 43DAG1/8D5, Novocastra) and 1:1000 for mouse anti-laminin α2 (MAB1922 Chemicon (Millipore)] for 1 h at room temperature, followed by incubation with Alexa Fluor 488 goat anti-mouse or donkey anti-sheep IgG antibodies (Molecular Probes) at 1:1000 dilution for 45 min at room temperature. All dilutions and washing steps were made in phosphate buffered saline. Immunostained sections were then mounted and observed with epifluorescence using a Nicon Eclipse E400 microscope. The fluorescent labeling was scored from 0 (absent) to 3 (intense) using a scale based on the proportion of positive fibers and intensity relative to the control muscle sections.

### Quantitative real-time PCR (qRT-PCR)

In order to assess the expression of *LARGE*, total RNA was extracted from liquid nitrogen fresh-frozen skeletal muscle tissues (3 mm^3^) from 26 patiens and 3 control samples using RNeasy® fibrous tissue mini kit (Qiagen) and cDNA was synthesized from 500 ng RNA using QuantiTect Reverse Transcription kit (Qiagen) according to manufacturer’s guidelines. Real-time PCR was then performed using SYBR Green JumpStart TaqReadyMix kit (Sigma) on Rotor-Gene 6000™ (Corbett Research) with the following conditions: 2 min at 94 °C, followed by 40 cycles of 5 s at 94 °C, 10 s at 58 °C, and 15 s at 72 °C. Reactions were performed in a total volume of 10 μL, including 5 μL SYBR Green JumpStart TaqReadyMix kit (Sigma), 1.2 μl of 25 mM MgCl_2_ (Sigma), 0.4 μl each of 10 mM forward and reverse primers and 1 μl of template cDNA. Mastermix with no cDNA was used as the no template control whereas water was used as the negative control. The relative amount of mRNA, normalized to an internal control human beta-actin *(ACTB)* and relative to a calibrator (control), was calculated by 2^-∆∆CT^. Primers used were as follows: *LARGE*-F: 5’-TGAGCCGTATGTTGTTGTGAGAC-3′; *LARGE*-R: 5’-GATGCGGTATTGCTTGTTGGAAC-3′ and *ACTB*-F: 5′- CGCAAAGACCTGTACGCCAAC -3′; *ACTB*-R: 5′- GAGCCGCCGATCCACACG -3′. All reactions were performed in triplicate and optimal threshold in 95–100% efficiency. All data was analyzed using Rotor-Gene 6000 Series Software 1.7 (Corbett Research).

### Statistical analysis

All experiments performed at least three times and data were evaluated as means ± SD. The significance of differences between experimental groups was determined using Mann-Whitney U test. *p* values less than 0.05 were considered statistically significant.

## Results

We assessed human muscle biopsies from DMD, BMD, sarcoglycanopathy, calpainopathy, dysferlinopathy, and merosin and collagen VI deficient CMDs, in addition to unclassified CMDs and MDs. Scoring for the severity of histopathological features and age at biopsy for each patient are shown in Table 1. In each muscular dystrophy group, samples had variable severity of histopathological findings such as necrosis, regeneration, fibrosis, adiposis and inflammation. Even some patients with the same diagnosis and similar age of biopsy showed different severity of pathological changes.

**Table 1:**
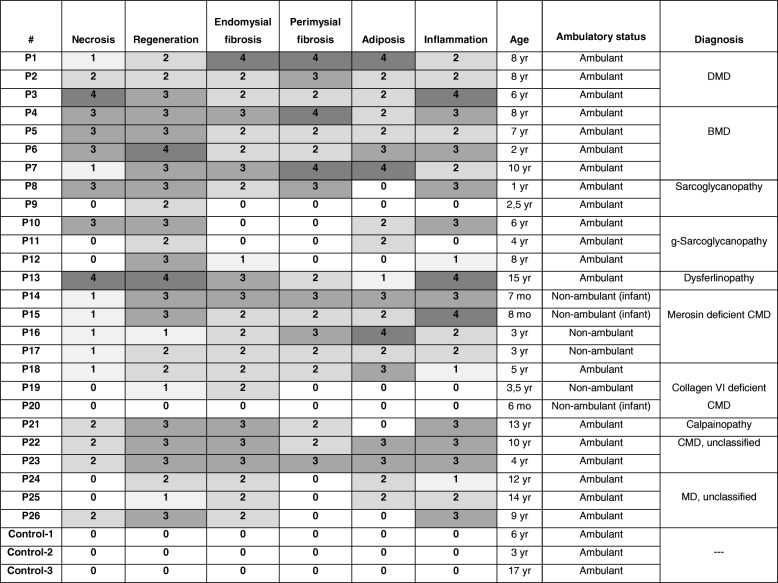
The clinicopathological diagnosis and histopathological characteristics of the patients (Assessment scores: 0: absent; 1: rare; 2: mild; 3: moderate; 4: severe)

**Fig. 1:**
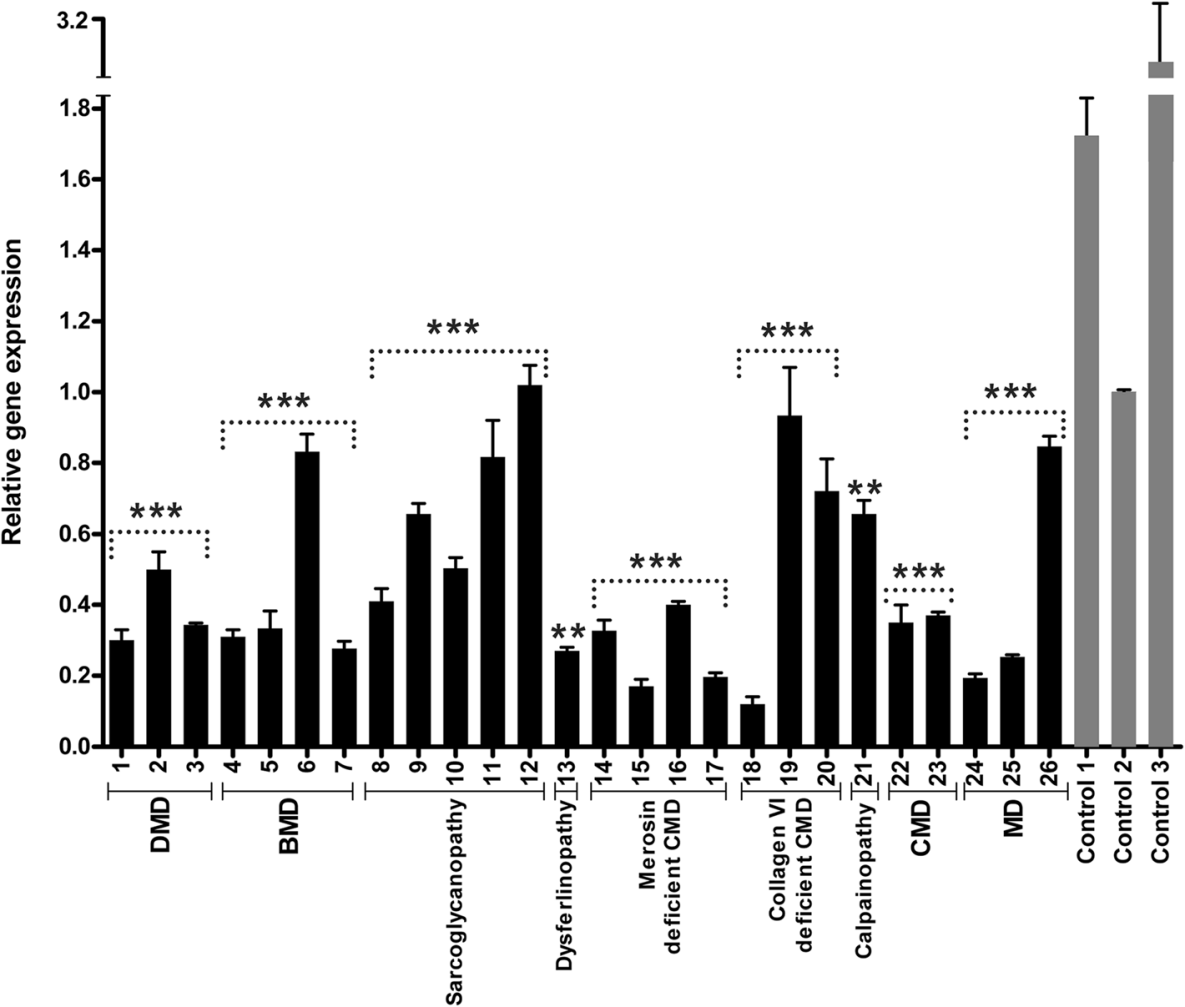
Relative expression levels of *LARGE,* measured by quantitative real time PCR in different types of muscular dystrophies. Bar graph representations are shown of the mean ± SEM values of the expression levels of patients (dark grey) and controls (light grey), respectively. Each bar represents the combined relative expression of three independent experiments measured by triplicate. Control 2 is used as calibrator. Error bars indicate standard deviations. Asterisks denote statistical significance as compared to the average of control group (Mann-Whitney U test, **p < 0.01, ***p < 0.001)

We first assessed the expression level of *LARGE* by qRT-PCR and detected reduced expression level of *LARGE* gene in analyzed samples, partly correlating with the form of muscular dystrophy (Fig. 1). The reduction of *LARGE* expression was more significant in all cases of DMD (P1, P2 and P3) and merosin deficient CMD (P14, P15, P16 and P17) as compared to control skeletal muscle. Also, *LARGE* mRNA levels were markedly reduced in all BMD cases, except one (P6). The expression of *LARGE* was similarly reduced in two cases of unclassified CMD (P22 and P23) and MD (P24 and P25), who had similar histopathological patterns. However, decreased expression in one case of Collagen VI deficient CMD (P18) with mild dystrophic features remains unexplained. The results of our study revealed different expression profiles for *LARGE* also among patients with different types of sarcoglycanopathies. Reduced *LARGE* expression levels were found also in the single dysferlinopathy (P13) and calpainopathy (P21) patients compared with age-matched healthy controls, with more severe reduction in *LARGE* and more severe dystrophic features in the case with dysferlin deficiency.

**Fig. 2:**
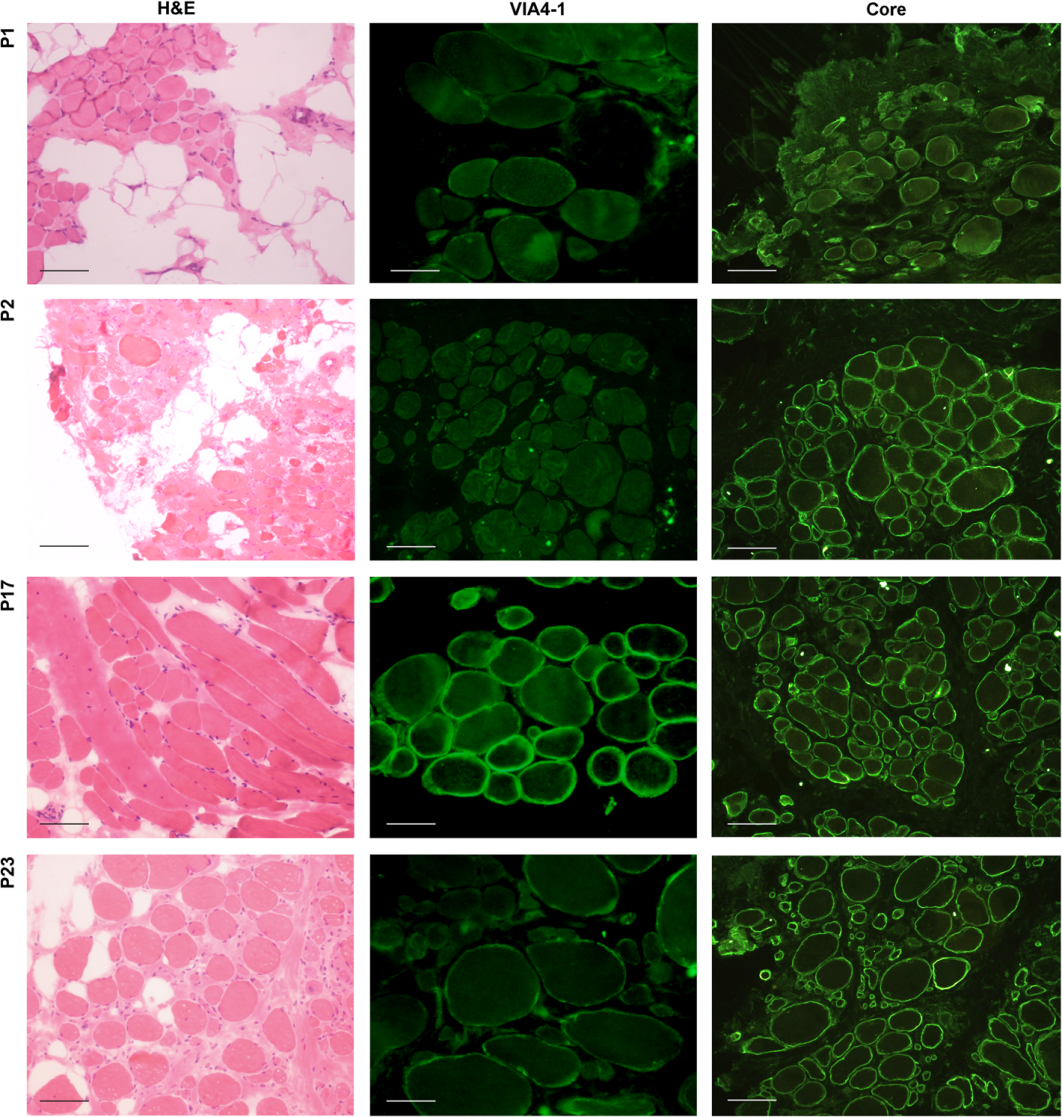
Illustrative images of muscle sections with hematoxylin and eosin staining (left-hand panel; scale bars: 40 μm.), glycosylated αDG labeling with VIA4–1 antibody (middle panel; scale bars: P1, P17 and P23: 20 μm.; P2: 40 μm.) and α-DG core antibody (right-hand panel; scale bars: 40 μm.). αDG labeling was normal in merosin deficiency (P17), mildly reduced in CMD (P23), but severely reduced/absent in cases with dystrophin deficiency (P1 and P2). Expression of the core αDG protein was well preserved in all samples

**Fig. 3:**
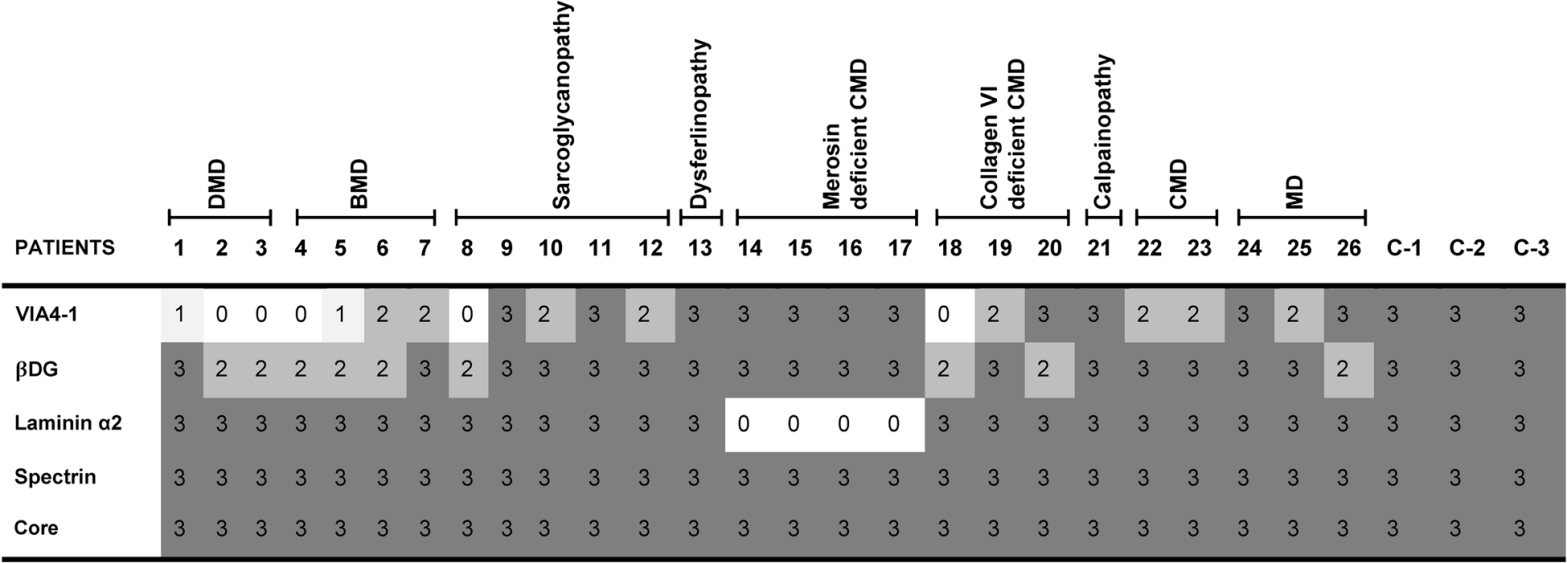
Representative results of immunofluorescence staining with VIA4–1, βDG, Laminin, Spectrin and αDG core antibodies. Immunolabeling scores were assigned to a scale of 0 to 3: (0 = absent; 1 = faint staining; 2 = moderate staining; 3 = intense staining)

In order to reveal a correlation between *LARGE* expression and functional glycosylation of αDG, skeletal muscle sections were immunostained using VIA4–1 antibody against fully glycosylated form of αDG, and evaluated together with αDG core protein (Fig. 2), beta-dystroglycan and laminin α2 immunostaining. The spectrum of VIA4–1 labeling was very variable and ranged from an absent to intense, compared with control skeletal muscle, whereas the immunoreactivity of core protein was indistinguishable between control and patients (Fig. 3). Therefore, the loss of VIA4–1 reactivity in some specimens reflects a reduction of α-DG glycosylation, rather than loss of α-DG core protein expression. The skeletal muscle of patients with DMD showed a marked depletion of αDG glycosylation. The reduction of VIA4–1 labeling in BMD group ranged from an absence on most fibres to a very mild reduction. Also, in some patients with sarcoglycanopathy, Collagen VI deficient CMD and unclassified MD, a mild reduction of sarcolemmal labelling with VIA4–1 antibody was detected. Interestingly, patient 8 and 18 displayed an almost total absence of αDG glycosylation relative to other patients within their group. Spectrin appeared normal in all cases indicating that the reduction in αDG hypoglycosylation was not due to non-specific damage of the plasma membrane. Except for merosin deficient CMDs, laminin α2 staining was also normal in all analyzed cases. Labeling of the transmembrane protein βDG was preserved in most cases, although a mild reduction has been reported in 9 of the 26 cases, mostly in dystrophinopathies. There was no significant relationship between reduction of *LARGE* expression and αDG hypoglycosylation, most obvious example being the merosin deficient CMD patients (P14, P15, P16 and P17) (Figs. 1 and 3) who showed normal immunostaining with αDG despite severe reduction of *LARGE* expression.

## Discussion

In order to determine correlations between *LARGE* expression, glycosylation of αDG and histopathological parameters, we analyzed *LARGE* gene expression profile and glycosylation status of αDG in various forms of muscular dystrophies, except for dystroglycanopathies. The expression level of *LARGE* was analyzed by qRT-PCR in skeletal muscle biopsies from 26 patients diagnosed as different forms of muscular dystrophy with various histopathological phenotypes (Table 1). Our findings indicated that most of the patients with severe histopathological phenotype (DMD and merosin deficient CMD) have decreased *LARGE* expression levels compared to control skeletal muscle (Fig. 1). We then evaluated whether there was a relationship between *LARGE* expression and αDG glycosylation. The functional glycosylation of αDG is associated with the binding of either IIH6 and/or VIA4–1 antibodies to αDG. Thus, immunofluorescence staining was done using the widely used VIA4–1 antibody. Our findings indicate that irrespective of the muscular dystrophy type, the degree of hypoglycosylation varied (Figs. 2 and 3) and there was generally no correlation with clinical/histopathological severity. Similar results have previously been reported in dystroglycanopathy patients with *Protein-O-mannosyl transferase 1 (POMT1), Protein-O-mannosyl transferase 2 (POMT2), POMGnT1*, *FKTN* and *FKRP* mutations [25]. Also, our results were not consistent with the findings which suggests a correlation between a reduction in αDG labeling and the clinical severity in mild, late-onset LGMD type 2I and severe CMD [20, 26]. On the other hand, we observed a severe reduction of αDG glycosylation in skeletal muscle biopsies of dystrophinopathy patients as a secondary finding, which has already been shown in DMD patients and mdx mouse [27, 28]. In contrast to those findings, the labelling obtained using the αDG core antibody was preserved in all patients, which suggested that the glycosylation of αDG is affected independently of DG protein expression.

In dystroglycanopathies, the effects of decreased glycosylation on laminin binding are variable [4, 11]. Therefore, further studies are necessary to evaluate if hypoglycosylation of αDG affects laminin binding or other functions of αDG in different types of muscular dystrophies other than dystroglycanopathy. Campbell and colleagues have previously demonstrated that short LARGE-glycan repeats reduces normal physiological function of muscle and predisposes it to dystrophy and showed that upregulation of *LARGE* and DG facilitates extension of LARGE-glycan repeat chains in differentiating mouse muscle [20]. However, consistent with our preliminary findings [29], we did not find any significant relationship between reduction of *LARGE* expression and αDG hypoglycosylation (Figs. 1 and 3), except for DMD. Our results suggest that LARGE enzyme may have an additional function in skeletal muscle fibers that is probably distinct from adding a critical sugar chain onto αDG. Equally likely is that the RNA changes in LARGE, while present, are not related at all to the MD phenotype. Transcriptional changes are difficult to interpret in prediction effects downstream.

## Conclusions

Glycosylation of αDG undergoes tissue and developmental stage specific changes, which indicates that it may have different roles in developing and fully differentiated skeletal muscle [4, 30]. αDG glycosylation is likely to be regulated by developmental and differential expression of numerous enzymes, including LARGE. Therefore the detected *LARGE* expression profiles in various form of muscular dystrophies may still reflect responses to pathological processes of skeletal muscle related to αDG glycosylation. Our cohort encompassed a wide range of muscular dystrophies, suggesting that a different pathomechanism may explain the relationship between *LARGE* expression and αDG glycosylation in the pathogenesis of different types of muscular dystrophies and highlighting the need for further studies.

## Abbreviations

*ACTB*: Beta-actin
BMD: Becker muscular dystrophy
CMD: Congenital muscular dystrophy
*DAG1*: DG-encoding gene
DG: Dystroglycan
DGC: Dystrophin-glycoprotein complex
DMD: Duchenne muscular dystrophy
ECM: Extracellular matrix
FCMD: Fukuyama congenital muscular dystrophy
*FKRP*: Fukutin-related protein
*FKTN*: Fukutin
H&E: Haematoxylin and eosin
*LARGE*: Like-acetylglucosaminyltransferase
LARGE-myd: myodystrophy mouse
LGMD: Limb-girdle muscular dystrophy
MEB: Muscle-eye-brain disease
*POMGNT1*: Protein-O-mannose ß2-N-acetylglucosaminyltransferase 1
*POMT1*: Protein-O-mannosyl transferase 1
*POMT2*: Protein-O-mannosyl transferase 2
qRT-PCR: Quantitative real-time PCR
WWS: Walker–Warburg syndrome
αDG: Alpha-dystroglycan
